# Comparison of tenecteplase vs. alteplase in addition to thrombectomy in patients with ischemic stroke caused by large vessel occlusion within 4.5 h: a network meta-analysis

**DOI:** 10.3389/fneur.2025.1730677

**Published:** 2026-01-06

**Authors:** Wenkui Li, Chuyue Wu, Li Li, Rong Deng

**Affiliations:** 1 Department of Neurology, Chongqing University Three Gorges Hospital, Chongqing, Wanzhou, China; 2 School of Medicine, Chongqing University, Chongqing, China; 3 Chongqing Municipality Clinical Research Center for Geriatric Diseases, Chongqing University Three Gorges Hospital, Chongqing, Wanzhou, China

**Keywords:** acute ischemic stroke, alteplase (rt-PA), meta analysis, tenecteplase (TNK), thrombectomy

## Abstract

**Background:**

The optimal reperfusion approach for acute ischemic stroke (AIS) due to large vessel occlusion (LVO) remains under debate. While endovascular thrombectomy (EVT) is the standard treatment, the role of intravenous thrombolysis before EVT—particularly with alteplase or tenecteplase—remains under investigation. This network meta-analysis (NMA) aimed to compare the efficacy and safety of three strategies: EVT alone, alteplase with EVT, and tenecteplase with EVT.

**Methods:**

A comprehensive search of Web of Science, PubMed, Cochrane Library and Embase was performed to find randomized controlled trials (RCTs) comparing the above interventions in AIS patients with LVO treated within 4.5 h of symptom onset. A Bayesian NMA framework was used to estimate pooled effects. The primary endpoint was Proportion of patients achieving a modified Rankin Scale (mRS) score of 0–2 at 90 days. Secondary endpoints included mRS 0–1, symptomatic intracerebral hemorrhage (sICH), and 90-day mortality.

**Results:**

Seven RCTs involving 2,793 patients were included. Among them, 1,290 received EVT alone, 1,124 alteplase with EVT, and 379 tenecteplase with EVT. Tenecteplase with EVT was associated with a higher rate of functional independence (mRS 0–2) at 90 days versus EVT alone (OR: 1.52; 95% CrI: 1.00–2.36), with the lower bound of the credible interval at the null. Tenecteplase with EVT also numerically outperformed alteplase plus EVT (OR: 1.48; 95% CrI: 0.97–2.36), although this difference was not statistically significant. No significant differences were observed among treatments in achieving excellent outcome (mRS 0–1), symptomatic intracerebral hemorrhage, or mortality. Tenecteplase plus EVT ranked highest in efficacy probability based on SUCRA values, but these rankings should be interpreted cautiously given the limited tenecteplase sample size and modest precision of the estimates.

**Conclusion:**

Tenecteplase with EVT may be associated with better 90-day functional outcomes than EVT alone and may offer advantages compared to alteplase with EVT for AIS patients with LVO treated within 4.5 h, without an observed excess risk of sICH or mortality. However, these findings are based on a small number of tenecteplase-treated patients and borderline credible intervals, so they should be interpreted cautiously and confirmed in larger, rigorously designed randomized trials.

**Systematic review registration:**

PROSPERO, identifier (CRD420251073350).

## Introduction

Acute ischemic stroke (AIS) is a major global health concern associated with significant morbidity, mortality, and long-term disability (1, 2). Rapid and effective reperfusion therapy is crucial for improving clinical outcomes in cases of large vessel occlusion (LVO), a prevalent and severe subtype. Endovascular thrombectomy (EVT) is the standard treatment for eligible patients with large vessel occlusion (LVO), offering better functional recovery than medical therapy alone (3–6).

Intravenous thrombolysis with alteplase continues to be commonly used within 4.5 h of symptom onset in AIS patients (7–11), however, its limited efficacy in achieving recanalization in large vessel occlusions has prompted growing interest in combination strategies. Intravenous thrombolysis followed by thrombectomy has been proposed to enhance early reperfusion, yet its incremental benefit over direct thrombectomy remains uncertain. Several randomized trials have reported inconsistent results regarding whether intravenous alteplase improves outcomes when administered prior to endovascular therapy (12–16). Notably, two studies reported no significant advantage of combining alteplase with EVT compared to EVT alone (13, 16), while three studies suggested equivalence between the two modalities (12, 14, 17). Tenecteplase has recently emerged as a promising alternative (18–24). Preliminary clinical trials have demonstrated encouraging signals, suggesting that tenecteplase may achieve higher rates of early reperfusion and improved functional outcomes in the pre-thrombectomy setting when compared to alteplase (25). However, these randomized trials were conducted in different settings, included patients with non-identical occlusion locations (two trials enrolling both anterior and posterior circulation strokes (25, 26), whereas the others focused on anterior circulation LVO (12–16)), and did not use fully uniform imaging or thrombolytic protocols (e.g., CT/CTA versus perfusion-based selection and low-dose alteplase in SKIP), which may introduce some heterogeneity into the available evidence.

Despite the growing body of literature evaluating these interventions, head-to-head comparisons among thrombectomy alone, plus alteplase with EVT, and tenecteplase with EVT remain limited. A network meta-analysis (NMA) is thus warranted to synthesize both direct and indirect evidence and to provide a comparative framework across multiple treatment arms. This methodology enables a more comprehensive assessment of the relative efficacy and safety of available reperfusion strategies.

This study aims to conduct a network meta-analysis to compare three reperfusion strategies for patients with AIS due to LVO: EVT alone, alteplase with EVT, and tenecteplase with EVT. The findings aim to clarify the comparative effectiveness of these approaches and support clinical decision-making in acute stroke treatment.

## Methods

This network meta-analysis followed the Preferred Reporting Items for Systematic Reviews and Meta-Analyses Extension for Network Meta-Analyses (PRISMA-NMA) guidelines (26). The study protocol was prospectively registered with PROSPERO (registration number: CRD420251073350).

### Search strategy and study selection

A thorough literature search was performed with four major databases: Web of Science, Cochrane Library PubMed, and Embase, covering all records up to May 2025. Only studies published in English were considered. The search strategy included keywords “endovascular thrombectomy,” “thrombolytic therapy,” “alteplase,” “tenecteplase,” and “ischemic stroke” (Supplementary Table 1).

Inclusion criteria were listed below: (1) Population: Adult patients diagnosed with acute ischemic stroke (AIS), treated within 4.5 h of symptom onset. (2) Interventions and Comparators: Trials comparing alteplase with EVT or tenecteplase with EVT, EVT alone were eligible. (3) Outcomes: Primary efficacy endpoint: Proportion of patients achieving a modified Rankin Scale (mRS) score of 0–2 at 90 days, indicating good functional recovery. Secondary outcomes: Incidence of symptomatic intracranial hemorrhage (sICH), proportion of patients with mRS 0–1 at 90 days, and mortality at 90 days. Study design: English-language randomized controlled trials (RCTs).

### Data extraction and quality assessment

Eligibility assessment was conducted through independent dual review of titles, abstracts, and full-text articles. A standardized data extraction form was employed to systematically capture information pertaining to study design, participant demographics, experimental interventions, control comparators, and reported outcome measures.

Methodological rigor was evaluated using the Cochrane Handbook’s domain-based framework for systematic reviews (27). A graphical representation of study quality was generated through Review Manager software (Version 5.3). The Confidence in Network Meta-Analysis (CINeMA) framework was employed to systematically evaluate the certainty of synthesized evidence across all outcome domains (28, 29).

### Data analysis

Network meta-analyses (NMAs) were implemented within a Bayesian hierarchical framework using R environment (version 4.2.0) with JAGS libraries. Markov chain Monte Carlo (MCMC) methods were employed for parameter estimation, utilizing non-informative priors across four parallel chains. Simulation parameters included 10,000 burn-in iterations, 20,000 adaptation steps, and 50,000 retained iterations for inference.

Convergence diagnostics were performed using trace plots, kernel density estimates, and the Gelman-Rubin potential scale reduction factor (PSRF), with PSRF values <1.05 indicating acceptable convergence (30). Model fit was assessed by comparing the posterior mean residual deviance to the number of unconstrained data points. Model selection followed deviance information criteria (DIC), where DIC > 5 denoted substantial superiority.

For dichotomous outcomes, treatment effects were estimated as pooled odds ratios (ORs) with corresponding 95% credible intervals (95% CrI) using binomial likelihood models with logit link functions (31). Statistical heterogeneity was evaluated through the Chi-squared test and quantified using I^2^ statistics, with non-significant heterogeneity defined as *p* ≥ 0.1 and I^2^ ≤ 50%.”

We conducted the analysis using the random effects model and considered the correlations among the effects of multiple groups of trials. This article also provides results for analysis using fixed-effects (FE) models (Supplementary Table 2). The transitivity assumption underlying the network meta-analysis was evaluated by comparing the distribution of clinical and methodological variables. In particular, we confirmed that all included trials enrolled patients with large vessel occlusion who were eligible for both intravenous thrombolysis and EVT, with intravenous thrombolysis administered within 4.5 h of symptom onset. Local inconsistency was evaluated using the node-splitting approach, with statistical significance defined as *p* < 0.05 for cross-validation of direct and indirect evidence comparisons (32). Treatment hierarchy was visualized through cumulative ranking curves (SUCRA plots) and accompanied by tabular summaries of rank probabilities.

## Results

**Figure 1:**
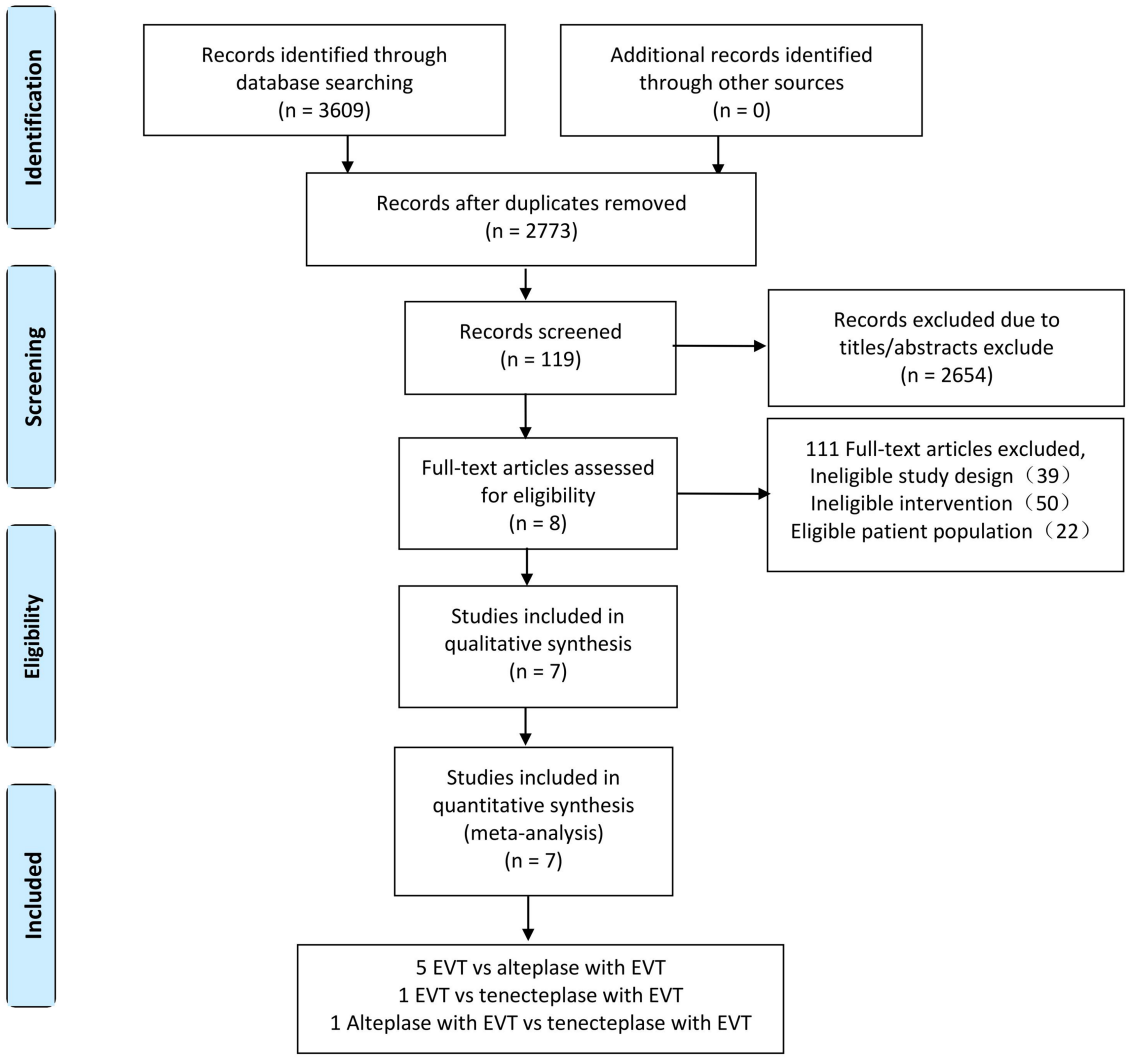
Flowchart illustrating the screening and research selection process of the meta-analysis. RCTs, randomised controlled trials.

A total of 3,609 studies were initially identified through the search. A total of 2,773 unique titles and abstracts were screened after eliminating duplicates. Subsequently, 119 articles underwent full-text evaluation, with reasons for exclusion detailed in Figure 1. Ultimately, eight articles comprising seven randomized controlled trials (RCTs) were deemed eligible for data extraction (Figure 1). The DIRECT-SAFE trial was excluded because it did not differentiate between bridging therapy with alteplase or tenecteplase (17). All included studies exhibited a high risk of selection bias owing to inadequate allocation concealment (Figure 1).

**Figure 2:**
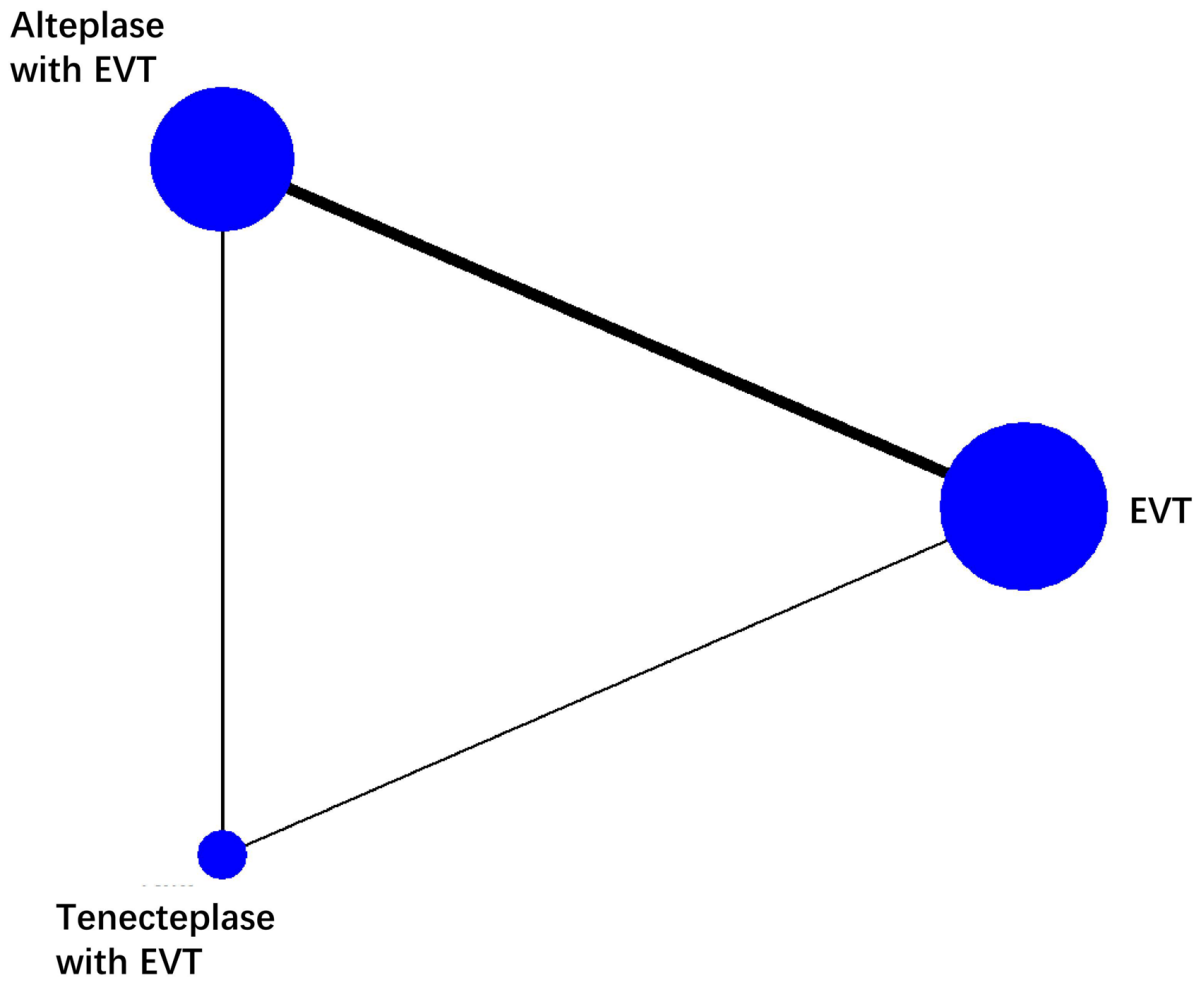
Network plot of each therapy in patients with acute ischemic stroke. The line width is proportional to the number of trials including each pair of treatments (direct comparison). The size of the circle is directly proportional to the total number of patients for each treatment in the network. EVT, endovascular thrombectomy.

We found 5 RCTs comparing EVT and alteplase with EVT, 1 comparing EVT and tenecteplase with EVT, 1 comparing the tenecteplase with EVT and alteplase with EVT. We analyzed data from 2,793 individuals: 1290 received EVT, 379 received tenecteplase with EVT, and 1,124 received alteplase with EVT. The characteristics of the included studies are summarized in the appendix (Table 1). Figure 2 illustrates the network diagram. The appendix (Supplementary Figure 2) provides the results of the heterogeneity test. Heterogeneity was absent for all outcomes except mortality, where significant heterogeneity was observed in the comparisons of tenecteplase with EVT versus EVT (I2 = 73.0%) and EVT plus alteplase versus EVT (I2 = 70.2%). The appendix (Supplementary Figure 3) provides the details of the consistency evaluation by node-splitting. DIC comparisons indicated negligible differences in fit between FE and RE consistency models; thus, RE model results are presented for all analyses (Supplementary Table 3).

**Table 1:** Characteristics of included studies.

Study	Design	Patient type	Intervention	Sample	Ages (mean, SD)	Males (%)	SICH	MRS 0–2	MRS 0–1	Mortality	Follow-up (months)
Campbell 2018 (33)	RCT	Anterior/posterior circulation	Tenecteplase with EVT	101	70.4(15.1)	57	1	65	52	10	3
			Alteplase with EVT	101	71.9(13.7)	51	1	52	43	18	
DIRECT-MT 2020 (16)	RCT	Anterior circulation	EVT	327	69(11.1)	57.8	14	119	80	58	3
			Alteplase with EVT	329	69(11.1)	55.0	20	121	74	62	
DEVT 2022 (13)	RCT	Anterior circulation	EVT	116	70(12.6)	56.9	5	63	44	20	3
			Alteplase with EVT	118	70(13.3)	55.9	7	55	37	21	
SKIP 2021 (14)	RCT	Anterior circulation	EVT	101	74(9.6)	55	8	60	41	8	3
			Alteplase with EVT	103	76(9.6)	70	2	59	46	9	
Fischer 2022 (12)	RCT	Anterior circulation	EVT	201	73(12.6)	48	5	114	80	22	3
			Alteplase with EVT	207	72(11.9)	50	7	135	89	17	
Qiu 2022 (17)	RCT	Anterior/posterior circulation	EVT	272	70(9.63)	58.5	18/269	120	76	54	3
			Tenecteplase with EVT	278	70(12.59)	57.9	23/271	147	97	62	
LeCouffe 2021 (15)	RCT	Anterior circulation	EVT	273	72(13.3)	59.0	16	134	44	56	3
			Alteplase with EVT	266	69(11.9)	54.1	14	136	41	42	

EVT, endovascular thrombectomy; RCT, randomized controlled study; NR, not reported; SICH, symptomatic intracranial hemorrhage.

### mRS 0–2 at 90 days

**Figure 3:**
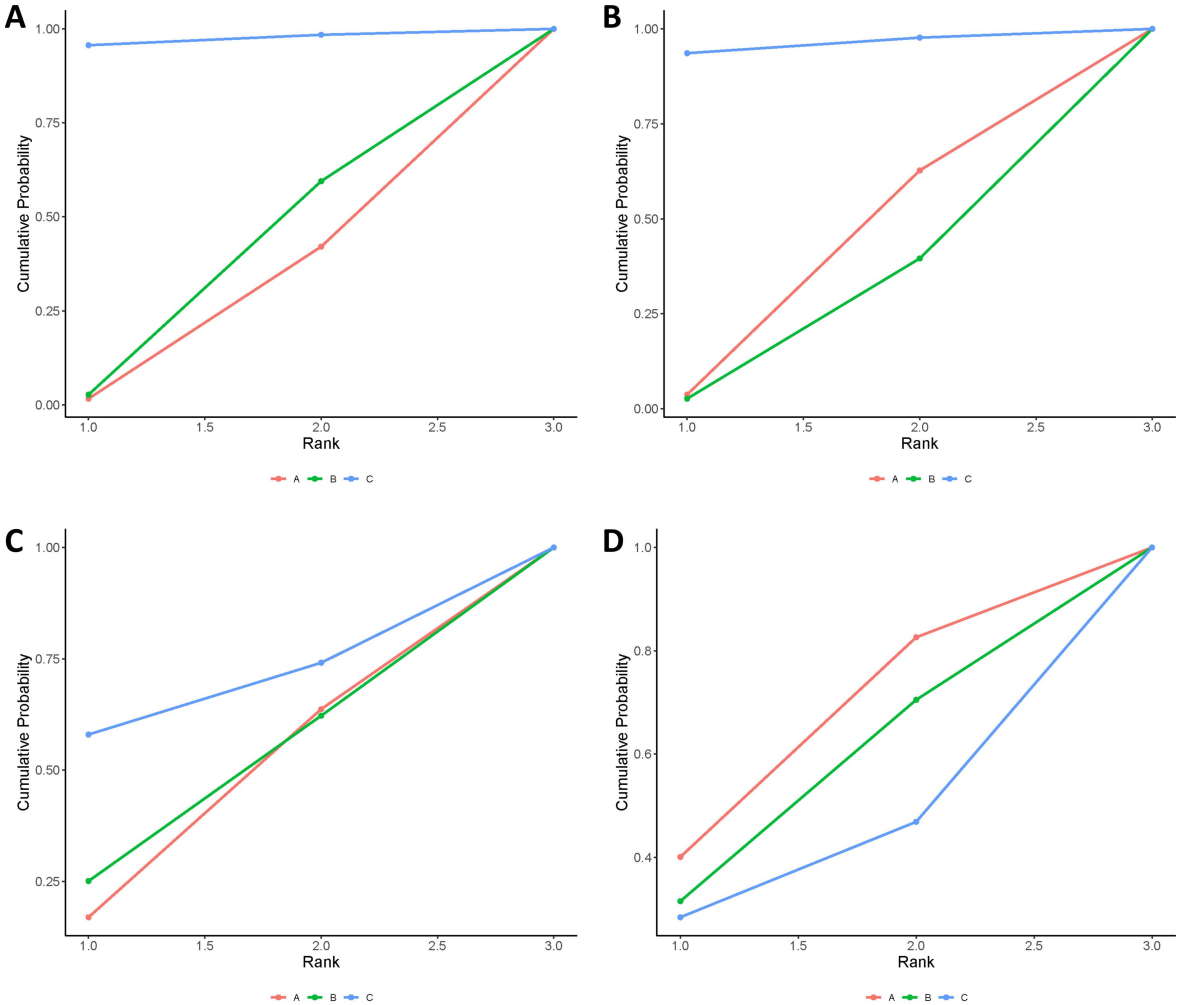
Bayesian ranking panel plots. **(A)** Proportion of patients achieving an mRS score of 0–2 at 90 days. **(B)** Proportion of patients achieving an mRS score of 0–1 at 90 days. **(C)** Incidence of symptomatic intracranial hemorrhage (sICH). **(D)** Death. Treatments: A, endovascular thrombectomy alone; B, alteplase with endovascular thrombectomy; C, tenecteplase with endovascular thrombectomy.

The combination of Tenecteplase and EVT was associated with a higher rate of 90-day functional independence (mRS 0–2) compared to EVT alone (OR: 1.52; 95% CrI: 1.00–2.36), with the lower bound of the credible interval lying at the null value, indicating borderline statistical significance and relatively weak evidence for a true benefit. Although tenecteplase with EVT demonstrated better efficacy than alteplase with EVT, this difference was not statistically significant (OR: 1.48; 95% CrI: 0.97–2.36). No statistically significant differences were identified between alteplase with EVT and EVT alone (Table 2). According to SUCRA values, tenecteplase with EVT ranked highest (SUCRA: 0.97), followed by alteplase with EVT (SUCRA: 0.32), and EVT alone (SUCRA: 0.21; Figure 3, Supplementary Table 4); however, SUCRA rankings reflect the relative probability of being the best treatment rather than definitive proof of superiority, and should be interpreted cautiously in the context of wide credible intervals and limited tenecteplase data.

**Table 2:** Estimates from a random-effects network model for all assessed outcomes up to 90 days.

Outcome measure	Estimates from NMA		
	OR (95% CrI)		
	Alteplase with EVT vs EVT	Tenecteplase with EVT vs EVT	Tenecteplase with EVT vs alteplase with EVT
mRS 0–2 at 90 days	1.03 (0.79, 1.30)	1.52 (1.00, 2.36)	1.48 (0.97,2.36)
mRS 0–1 at 90 days	0.97 (0.77, 1.21)	1.38 (0.94, 2.03)	1.43 (0.96, 2.15)
sICH	1.01 (0.50, 1.91)	1.25 (0.36, 4.16)	1.23 (0.33, 4.77)
Death	0.97 (0.68, 1.42)	0.90 (0.46, 1.58)	0.93 (0.45, 1.67)

CrI, credible interval; EVT, endovascular thrombectomy; NMA, network meta-analysis; OR, odds ratio; sICH, symptomatic intracerebral hemorrhage.

### mRS 0–1 at 90 days

The treatments did not show any statistically significant differences. However, tenecteplase with EVT showed numerically higher rates of achieving an excellent outcome (mRS 0–1) at 90 days compared to EVT alone or alteplase with EVT (Table 2). SUCRA analysis confirmed tenecteplase with EVT as having the highest probability of achieving mRS 0–1 (SUCRA: 0.96; Figure 3, Supplementary Table 4); but, similar to the primary outcome, these rankings should be viewed as probabilistic rather than conclusive, especially given the imprecision of the effect estimates and the small number of tenecteplase-treated patients.

### Symptomatic intracerebral hemorrhage (sICH)

No significant differences were found among the treatments regarding symptomatic intracerebral hemorrhage (Table 2). According to SUCRA values, tenecteplase combined with EVT presented the highest relative risk (SUCRA 0.68), while EVT alone showed the lowest risk (SUCRA: 0.39; Figure 3, Supplementary Table 4); and, as noted above, SUCRA rankings should be viewed as probabilistic rather than definitive.

### Death

The treatments showed no significant difference in mortality risk (Table 2). EVT alone had the highest SUCRA value for mortality (SUCRA: 0.61), indicating numerically higher mortality probability, followed by alteplase with EVT (SUCRA: 0.51), and tenecteplase with EVT (SUCRA, 0.38; Figure 3, Supplementary Table 4); but the SUCRA-based hierarchy should be interpreted cautiously.

### Certainty of evidence

The results of assessment of the confidence are shown in the appendix (Supplementary Table 5). The certainty of evidence for the all outcomes was consistently low to very low across all comparisons.

## Discussion

In this network meta-analysis, we observed that bridging therapy using intravenous tenecteplase followed by EVT was associated with a significantly higher rate of functional independence (mRS 0–2) at 90 days compared with EVT alone (OR: 1.52; 95% CrI: 1.00–2.36). Tenecteplase bridging therapy also showed a tendency toward greater efficacy compared to alteplase bridging, though this advantage did not reach statistical significance. Importantly, no significant differences were found among the three treatment strategies regarding excellent outcomes (mRS 0–1), sICH, or mortality. These comparative estimates for tenecteplase should, however, be interpreted with caution given the relatively small number of tenecteplase-treated patients and the between-trial differences in design and enrolled populations.

Our findings are broadly consistent with emerging evidence suggesting that tenecteplase may have certain advantages over alteplase in AIS treatment strategies involving bridging therapy, including improved pharmacokinetics, higher fibrin specificity, and ease of administration via single bolus (25, 33). The SUCRA values for tenecteplase combined with EVT (0.97 for mRS 0–2 and 0.96 for mRS 0–1) highlight its potential clinical benefits, but these findings should be interpreted cautiously due to the limited number of direct comparative studies and the reliance on indirect comparisons within our network meta-analysis.

Consistent with our results, recent studies have provided mixed outcomes concerning bridging therapy versus EVT alone. For instance, the DIRECT-MT and DEVT trials demonstrate non-inferiority of EVT alone compared to EVT with alteplase (13, 16). Similarly, the randomized controlled trial conducted by LeCouffe et al. which enrolled 539 European patients with anterior circulation LVO, reported no significant difference in 90-day disability outcomes or rates of sICH between EVT alone and alteplase plus EVT (15). Interestingly, while some studies reported a higher reperfusion rate in the alteplase-bridging group compared to ECT alone, this did not always translate into better clinical outcomes (12). A recent meta-analysis of six studies also failed to establish the non-inferiority of EVT alone compared with combined thrombolysis and EVT (34). Likewise, an individual patient data meta-analysis found no improvement in functional outcomes with bridging therapy (alteplase with EVT) in patients with carotid tandem lesions, reinforcing the ongoing uncertainty regarding the role of intravenous thrombolysis before EVT (35). Although combined intravenous thrombolysis and mechanical thrombectomy could theoretically be disadvantaged by a delay in initiating thrombectomy due to the preparation of thrombolytic therapy, the randomization-to-puncture times did not differ significantly between the two groups (14). Moreover, these mixed results arise from trials that differ in baseline infarct core size, collateral circulation, clot location and workflow times, factors that could not be standardised or fully accounted for in our aggregate-level analysis.

Importantly, our findings support the growing interest in tenecteplase as a thrombolytic agent in AIS. A meta-analysis of 38 observational studies found that combining intravenous thrombolysis with EVT increased the likelihood of achieving functional independence at 3 months compared to EVT alone (17). Although only one RCT in our analysis directly compared tenecteplase with alteplase before EVT, it suggested a trend toward better outcomes with tenecteplase (33). In addition, a recent RCT demonstrated that patients received intravenous tenecteplase was higher rate of functional independence at 90 days compared with EVT alone (25). The total number of tenecteplase-treated patients across these trials remains modest, and the current evidence base remains limited, emphasizing the need for larger, rigorously designed head-to-head trials to clarify tenecteplase’s role in bridging therapy.

Safety remains a critical consideration in thrombolytic strategies. Although no significant differences in sICH were identified, our SUCRA analysis indicated a slightly elevated hemorrhage risk with tenecteplase bridging compared to EVT alone. While this difference was not statistically significant, clinicians should remain vigilant about hemorrhagic complications, especially in high-risk populations. It should be noted that definitions of sICH were not fully uniform across trials. For example, DEVT (13) and Qiu et al. (25)^.^ defined sICH as symptomatic intracerebral hemorrhage occurring within 48 h, whereas Campbell et al. (33). used narrower time windows for ascertainment (e.g., within 36 h after treatment), which may have influenced the comparability of pooled safety estimates.

This study possesses several strengths. First, we included only randomized controlled trials to ensure methodological rigor and minimize bias. The target population was carefully selected—patients with LVO within 4.5 h of symptom onset and meeting both thrombolysis and thrombectomy eligibility criteria. Despite the limited number of studies directly comparing EVT alone, EVT plus tenecteplase, and EVT plus alteplase, the network meta-analytic approach enabled integration of both direct and indirect comparisons, yielding consistent and robust results.

However, it is important to recognize certain limitations. The number of available RCTs evaluating tenecteplase was small, which may affect the stability of comparative estimates. Moreover, SKIP (14) investigated alteplase at a reduced dose of 0.6 mg/kg, whereas the other included trials used the standard 0.9 mg/kg regimen (12, 13, 15, 16), which may limit the extrapolation of our alteplase estimates beyond the specific dosing schedules evaluated. Furthermore, heterogeneity in enrolled populations—particularly regarding occlusion site (anterior vs. posterior circulation) and ethnicity (e.g., Asian vs. European cohorts)—may limit the generalizability of our findings, and the trials also differed in imaging strategies (e.g., CT/CTA or MRA), thrombectomy techniques and devices, timing of EVT, and non-uniform definitions of sICH. All included studies were open-label, which may introduce performance and detection biases. Additionally, individual patient-level data were unavailable, restricting the possibility of subgroup or adjusted analyses. Several treatment contrasts, particularly those involving tenecteplase, relied largely on indirect evidence, so the assumptions of transitivity and consistency cannot be fully verified and the precision of treatment rankings is limited. Finally, although we performed an extensive literature search, some relevant unpublished or gray literature may have been missed, and evaluation of publication bias in network meta-analysis remains challenging due to the limited number of comparisons per treatment dyad.

Our network meta-analysis indicates that tenecteplase bridging therapy before EVT could lead to better functional outcomes than EVT alone and may be more effective than alteplase bridging therapy for AIS patients with LVO within 4.5 h of symptom onset. Although promising, these findings require confirmation through large-scale, prospective randomized trials before tenecteplase can be definitively recommended as the preferred thrombolytic agent in bridging therapy.

## Conclusion

Our network meta-analysis suggests that tenecteplase bridging therapy before EVT may be associated with improved 90-day functional independence in AIS patients with LVO within 4.5 h of symptom onset, compared to EVT alone and potentially surpassing alteplase bridging therapy. However, these signals are based on a limited number of tenecteplase-treated patients, borderline credible intervals and predominantly indirect comparisons, and should therefore be interpreted with caution. Additional large-scale randomized trials are necessary to confirm these findings and develop conclusive treatment guidelines.

## Data Availability

The datasets presented in this study can be found in online repositories. The names of the repository/repositories and accession number(s) can be found in the article/Supplementary material.
