# Supplementary material for: Comparison of tenecteplase vs. alteplase in addition to thrombectomy in patients with ischemic stroke caused by large vessel occlusion within 4.5 h: a network meta-analysis
Source: Front Neurol. 2026 Jan 6;16:1730677. doi: 10.3389/fneur.2025.1730677 (PMC12815846; doi:10.3389/fneur.2025.1730677)
Supplement: Supplementary file 1 [file Table_1.docx]

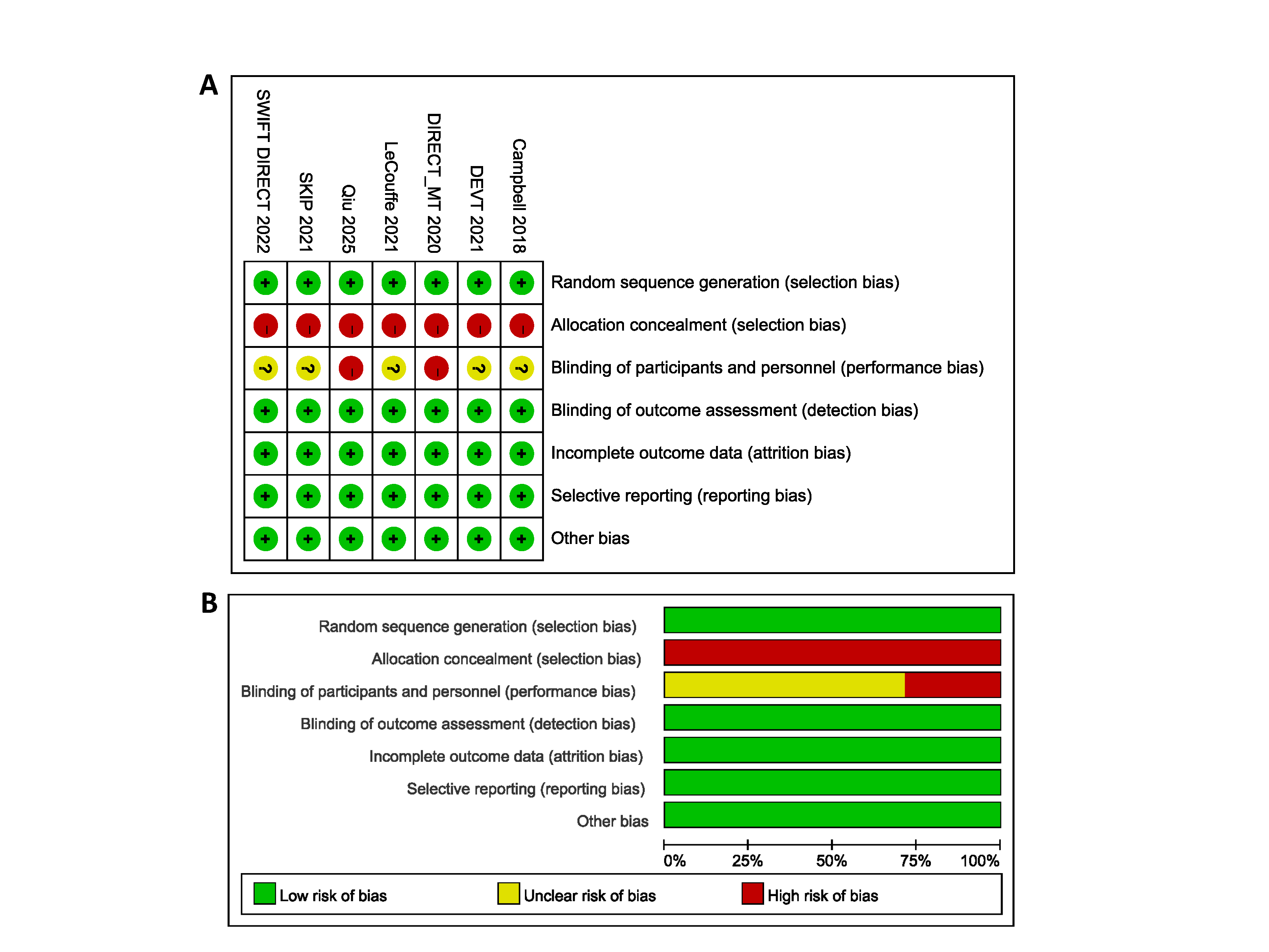


**Supplementary Figure 1 All included studies exhibited a high risk of selection bias owing to inadequate allocation concealment**

(A) Risk of Bias Summary: Review authors’ assessments of each risk of bias domain for every included study.

(B) Risk of Bias Graph: Review authors’ assessments of each risk of bias domain presented as the percentage of studies judged at each level of risk.


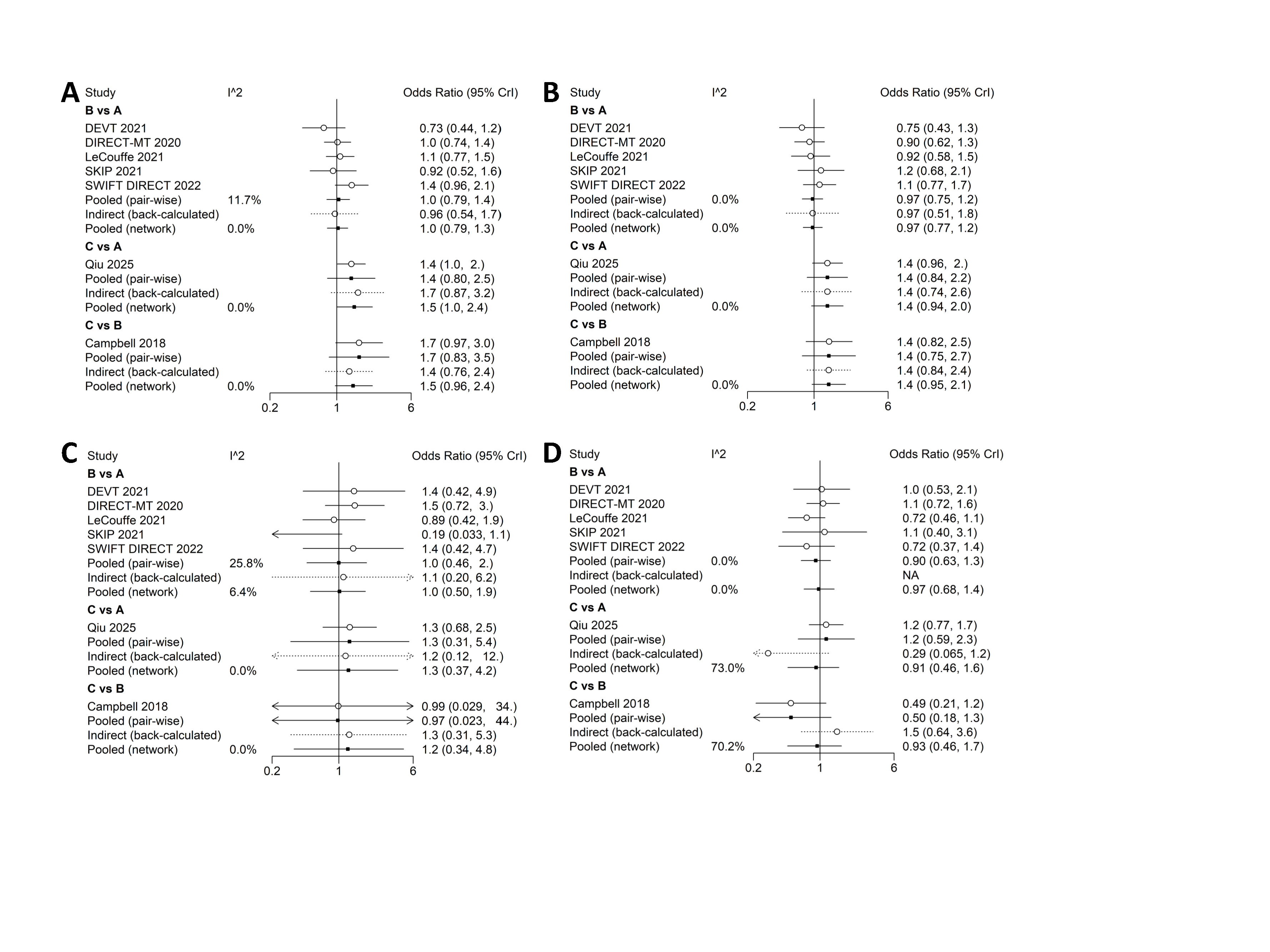


**Supplementary Figure 2 Results of Heterogeneity Testing**

(A) Proportion of patients achieving a modified Rankin Scale (mRS) score of 0–2 at 90 days.

(B) Proportion of patients achieving an mRS score of 0–1 at 90 days.

(C) Incidence of symptomatic intracerebral hemorrhage (sICH).

(D) mortality.

treatment A: endovascular thrombectomy alone; treatment B: alteplase with endovascular thrombectomy; treatment C: tenecteplase with endovascular thrombectomy. CrI: credible interval.


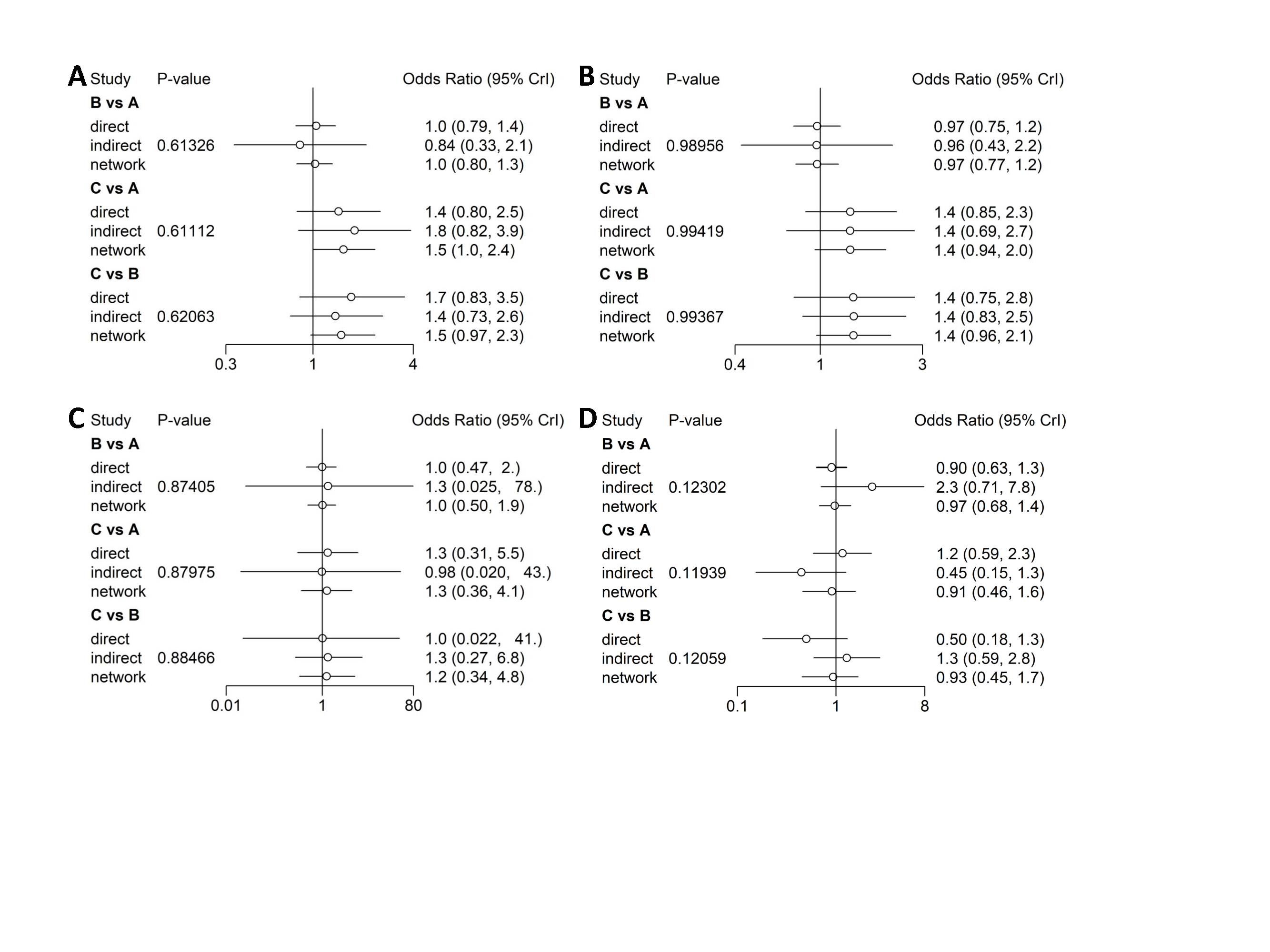


**Supplementary Figure 3 Results of Consistency Assessment Using Node-Splitting Analysis**

(A) Proportion of patients achieving a modified Rankin Scale (mRS) score of 0–2 at 90 days.

(B) Proportion of patients achieving an mRS score of 0–1 at 90 days.

(C) Incidence of symptomatic intracerebral hemorrhage (sICH).

(D) mortality

treatment A: endovascular thrombectomy alone; treatment B: alteplase with endovascular thrombectomy; treatment C: tenecteplase with endovascular thrombectomy. CrI: credible interval.
