# Supplementary material for: Comparison of tenecteplase vs. alteplase in addition to thrombectomy in patients with ischemic stroke caused by large vessel occlusion within 4.5 h: a network meta-analysis
Source: Front Neurol. 2026 Jan 6;16:1730677. doi: 10.3389/fneur.2025.1730677 (PMC12815846; doi:10.3389/fneur.2025.1730677)
Supplement: Supplementary file 2 [file Table_2.docx]

**Supplementary Table 1 Comprehensive Description of the Literature Search Strategy**

| #1 | (Cerebral infarction):ab,ti,kw OR (Infarction, Cerebral):ab,ti,kw OR(Cerebral Infarct):ab,ti,kw OR(Infarct, Cerebral):ab,ti,kw OR(Subcortical Infarction):ab,ti,kw OR(Infarction, Subcortical):ab,ti,kw OR(Subcortical Infarctions):ab,ti,kw OR(Posterior Choroidal Artery Infarction):ab,ti,kw OR(Anterior Choroidal Artery Infarction):ab,ti,kw OR(Ischemic Stroke):ab,ti,kw OR(Stroke, Ischemic):ab,ti,kw OR(Ischaemic Stroke):ab,ti,kw OR(Stroke, Ischaemic):ab,ti,kw OR(Cryptogenic Embolism Stroke):ab,ti,kw OR(Cryptogenic Embolism Strokes):ab,ti,kw OR(Embolism Stroke, Cryptogenic):ab,ti,kw OR(Stroke, Cryptogenic Embolism):ab,ti,kw OR(Wake-up Stroke):ab,ti,kw OR(Stroke, Wake-up):ab,ti,kw OR(Wake up Stroke):ab,ti,kw |
| --- | --- |
| #2 | (Tissue Plasminogen Activator):ab,ti,kw OR (Plasminogen Activator, Tissue):ab,ti,kw OR(Tisokinase):ab,ti,kw OR(Tissue-Type Plasminogen Activator):ab,ti,kw OR(Tissue Type Plasminogen Activator):ab,ti,kw OR(TTPA):ab,ti,kw OR(T-Plasminogen Activator):ab,ti,kw OR(Alteplase):ab,ti,kw OR(tenecteplase ):ab,ti,kw OR(Metalyse):ab,ti,kw OR(TNKase):ab,ti,kw OR(Thrombolytic Therapy):ab,ti,kw OR(Therapeutic Thrombolysis):ab,ti,kw OR(Therapeutic Thrombolyses):ab,ti,kw OR(Thrombolyses, Therapeutic):ab,ti,kw OR(Thrombolysis, Therapeutic):ab,ti,kw OR(Therapy, Fibrinolytic):ab,ti,kw OR(Fibrinolytic Therapies):ab,ti,kw OR(Therapies, Fibrinolytic):ab,ti,kw OR(Therapy, Thrombolytic):ab,ti,kw OR(Therapies, Thrombolytic):ab,ti,kw OR(Thrombolytic Therapies):ab,ti,kw OR(Fibrinolytic Therapy):ab,ti,k |
| #3 | (randomized controlled trial):ab,ti,kw OR(randomized):ab,ti,kw OR(placebo):ab,ti,kw OR(RCT):ab,ti,kw |
| #4 | #1 and #2 and #3 |

**Supplementary Table 2 Estimates from a Fixed-Effects Network Model for All Assessed Outcomes up to 90 Days**

| **Outcome measure** | **Estimates from NMA** | | |
| --- | --- | --- | --- |
|  | **OR (95% CrI)** | | |
|  | **Alteplase with EVT vs EVT** | **Tenecteplase with EVT vs EVT** | **Tenecteplase with EVT vs alteplase with EVT** |
| mRS 0–2 at 90 days | 1.04 (0.87, 1.23) | 1.51 (1.12, 2.02) | 1.45 (1.06, 1.99) |
| mRS 0–1 at 90 days | 0.97 (0.80, 1.17) | 1.38 (1.01, 1.88) | 1.43 (1.03, 2.00) |
| sICH | 1.05 (0.7, 1.56) | 1.29 (0.69, 2.44) | 1.23 (0.59, 2.59) |
| Death | 0.96 (0.76, 1.22) | 0.97 (0.67, 1.40) | 1.01 (0.67, 1.52) |

CrI: credible interval; EVT: endovascular thrombectomy; NMA: network meta-analysis; OR: odds ratio. sICH: symptomatic intracerebral hemorrhage

**Supplementary Table 3 Summary of Model Fit Statistics for Fixed-Effects and Random-Effects Network Meta-Analyses by Outcome**

| **Model** | **Number of data points** | **Posterior total**  **residual deviance** | **DIC** |
| --- | --- | --- | --- |
| **Functional independence** | | | |
| FE consistency | 14 | 9.03 | 22.99 |
| RE inconsistency | 14 | 11.79 | 25.49 |
| RE consistency | 14 | 10.96 | 24.28 |
| **Disabilityfree** | | | |
| FE consistency | 14 | 9.00 | 20.26 |
| RE inconsistency | 14 | 11.07 | 23.47 |
| RE consistency | 14 | 10.23 | 21.89 |
| **Symptomatic intracranial hemorrhage** | | | |
| FE consistency | 14 | 9.13 | 24.18 |
| RE inconsistency | 14 | 12.37 | 27.20 |
| RE consistency | 14 | 11.18 | 25.06 |
| **Mortality** | | | |
| FE consistency | 14 | 9.02 | 24.20 |
| RE inconsistency | 14 | 11.38 | 23.77 |
| RE consistency | 14 | 11.19 | 24.75 |

FE fixed-effects. RE random-effects. DIC: deviance information criteria.

**Supplementary Table 4 SUCRA of competing treatments for patients with acute large-vessel occlusion**

| **Outcomes** | **A** | **B** | **C** |
| --- | --- | --- | --- |
| Good functional outcome | 0.21 | 0.32 | 0.97 |
| Excellent functional outcome | 0.34 | 0.21 | 0.96 |
| Symptomatic intracranial hemorrhage | 0.39 | 0.43 | 0.68 |
| Mortality | 0.61 | 0.51 | 0.38 |

Surface under the cumulative ranking curve (SUCRA) was expressed as a percentage. A: endovascular thrombectomy alone; B: alteplase with endovascular thrombectomy; C: tenecteplase with endovascular thrombectomy.

**Supplementary Table 5 Certainty of evidence**

| **Comparison** | **Number of studies** | **Within-study bias** | **Reporting bias** | **Indirectness** | **Imprecision** | **Heterogeneity** | **Incoherence** | **Confidence rating** |
| --- | --- | --- | --- | --- | --- | --- | --- | --- |
| **mRS 0–2 at 90 days** | | | | | | | | |
| C vs B | 1 | Some concerns | Low risk | No concerns | No concerns | Some concerns | No concerns | Low |
| C vs A | 5 | Some concerns | Low risk | No concerns | No concerns | Some concerns | No concerns | Low |
| A vs B | 1 | Some concerns | Low risk | No concerns | No concerns | Some concerns | No concerns | Low |
| **Symptomatic intracranial hemorrhage** | | | | | | | | |
| C vs B | 1 | Some concerns | Low risk | No concerns | Major concerns | No concerns | No concerns | Very low |
| C vs A | 5 | Some concerns | Low risk | No concerns | Major concerns | No concerns | No concerns | Very low |
| A vs B | 1 | Some concerns | Low risk | No concerns | Major concerns | No concerns | No concerns | Very low |
| **mRS 0–1 at 90 days** | | | | | | | | |
| C vs B | 1 | Some concerns | Low risk | No concerns | No concerns | Some concerns | No concerns | Low |
| C vs A | 5 | Some concerns | Low risk | No concerns | Some concerns | Some concerns | No concerns | Very low |
| A vs B | 1 | Some concerns | Low risk | No concerns | No concerns | Some concerns | No concerns | Low |
| **Death** | | | | | | | | |
| C vs B | 1 | Some concerns | Low risk | No concerns | Major concerns | No concerns | Some concerns | Very low |
| C vs A | 5 | Some concerns | Low risk | No concerns | Major concerns | No concerns | Major concerns | Very low |
| A vs B | 1 | Some concerns | Low risk | No concerns | Major concerns | No concerns | Some concerns | Very low |

A: Alteplase with endovascular thrombectomy; B:Tenecteplase with endovascular thrombectomy; C: endovascular thrombectomy.
